# Primed atypical ductal hyperplasia-associated fibroblasts promote cell growth and polarity changes of transformed epithelium-like breast cancer MCF-7 cells via miR-200b/c-IKKβ signaling

**DOI:** 10.1038/s41419-017-0133-1

**Published:** 2018-01-26

**Authors:** Yan Sun, Dan Yang, Lei Xi, Yanlin Chen, Lixin Fu, Kexin Sun, Jiali Yin, Xiaotian Li, Shuiqing Liu, Yilu Qin, Manran Liu, Yixuan Hou

**Affiliations:** 1 Key Laboratory of Laboratory Medical Diagnostics, Chinese Ministry of Education, Chongqing Medical University, #1 Yi-Xue-Yuan Road, 400016 Chongqing, Yu-zhong District, China; 2 Department of Cell Biology and Medical Genetics, Basic Medical School, Chongqing Medical University, #1 Yi-Xue-Yuan Road, 400016 Chongqing, Yu-zhong District, China; 3 Department of Endocrine and Breast Surgery, The First Affiliated Hospital of Chongqing Medical University, #1 You-Yi Road, 400016 Chongqing, Yu-zhong District, China; 4 Experimental Teaching Center of Basic Medicine Science, Chongqing Medical University, #1 Yi-Xue-Yuan Road, 400016 Chongqing, Yu-zhong District, China

## Abstract

Cancer-associated fibroblasts (CAFs) support tumorigenesis by stimulating cancer cell proliferation, and invasion, but how the premalignant stromal fibroblasts trigger epithelial changes remain unclear. We demonstrate that atypical ductal hyperplasia-associated fibroblasts (AHFs) are one kind of activated fibroblasts and stimulate cell growth and polarity change of epithelium-like tumor cell MCF-7 as CAFs-like fibroblasts. Microarray shows miR-200b and miR-200c are downregulated during AHFs and CAFs, and contribute to stromal fibroblast activity. Additionally, miR-200b/c with target gene IKKβ (inhibitor of nuclear factor kappa-B kinase β) control PAI-1 (plasminogen activator inhibitor-1) expression to regulate growth and polarity changes of MCF-7 cells through NF-κB pathway. Exploring the difference of AHFs in premalignant transformation is crucial for understanding the pathobiology of breast cancer progression.

## Introduction

Benign breast disease is an important risk factor for breast cancer^1^. The currently working hypothesis of breast cancer initiation suggests that breast cancer evolves in a linear progression through sequential stages of hyperplastic benign breast lesions; atypical hyperplasia (AH, including atypical ductal hyperplasia (ADH) and/or atypical lobular hyperplasia); carcinoma in situ (e.g., ductal carcinoma in situ (DCIS) and/or lobular carcinoma in situ); and, ultimately, invasive breast cancer (IBC)^2–4^. Although considerable progress has been made in elucidating the genetic events in noninvasive and IBC, the relationship between premalignant and in situ lesions is not completely established^5^. As the ductal type lesions encompass almost 80% of all diagnosed breast cancers^6^, ADH are derived from outgrowths of luminal epithelial cells and are morphologically related to low-grade DCIS. Accordingly, ADH is a good model to simulate breast carcinoma initiation^7^. However, it remains unclear what drives malignant transformation of ADH and what is the potential molecular mechanism.

It is established that most tumors follow the activation of tumor microenvironment remodeling, and reactive microenvironment induces the malignant cells to proliferate, migrate, and invade^8,9^. Fibroblasts are a major cell type of microenvironment and cancer-associated fibroblasts (CAFs) are thought to favor tumor progression, including breast tumor progression^10^. CAFs differ from normal fibroblasts (NFs) in phenotypic properties, the expression of growth factors, and the molecule synthesis of the extracellular matrix (ECM)^11^. When CAFs mixed with mammary epithelial cells, they can induce a faster tumor growth than NFs^12^. Whereas, at a critical time during premalignant transformation, whether the premalignant fibroblasts trigger epithelial changes “priming” or provoking the premalignant tumor are intriguing for understanding the pathobiology of cancer progression, but these questions are not well understood^12,13^. ADH as a good model of premalignant breast tumor, fibroblasts in ADH (AHFs) may play a specific role in the premalignant progression. However, whether the AHFs exist between CAFs and NFs and whether AHFs trigger epithelial changes during cell transformation of malignancy process are unclear.

MicroRNAs (miRNAs) are small noncoding RNAs that suppress the translation of target messenger RNA (mRNA) depending on the complementarity between miRNA and the 3′-untranslated region (3′-UTR) of target mRNA^14^. Our previous studies have showed downregulated miR-200 family in CAFs contribute to breast cancer cell invasion and ECM remodeling^9,15^. Although extensive miRNAs research has been conducted on CAFs, few works are known about the miRNA functions in the fibroblasts of premalignant lesions.

In this study, it was examined whether AHFs contribute distinctive microenvironment influences on breast tumor cells and miRNA in AHFs and CAFs plays a role for malignant transformation. Our data show that AHFs are a kind of activated fibroblasts in ADH, which have a distinctive biological potential to stimulate cell growth and polarity changes for epithelium-like breast cancer cells. Furthermore, the downregulated miR-200b/c in AHFs and CAFs contribute to the activation of fibroblasts by targeting IKKβ (inhibitor of nuclear factor kappa-B kinase β) and stimulating NF-κB pathway. PAI-1 (plasminogen activator Inhibitor-1), the downstream target of NF-κB in AHFs and CAFs, acts as a core in trigging cell growth and polarity changes of epithelium-like tumor cell MCF-7. Therefore, our works provide a novelty insight into our knowledge for fibroblasts in premalignant to promote malignant transformation of mammary epithelium.

## Result

### Activated fibroblasts exist in mammary atypical hyperplasia tissues

**Fig. 1:**
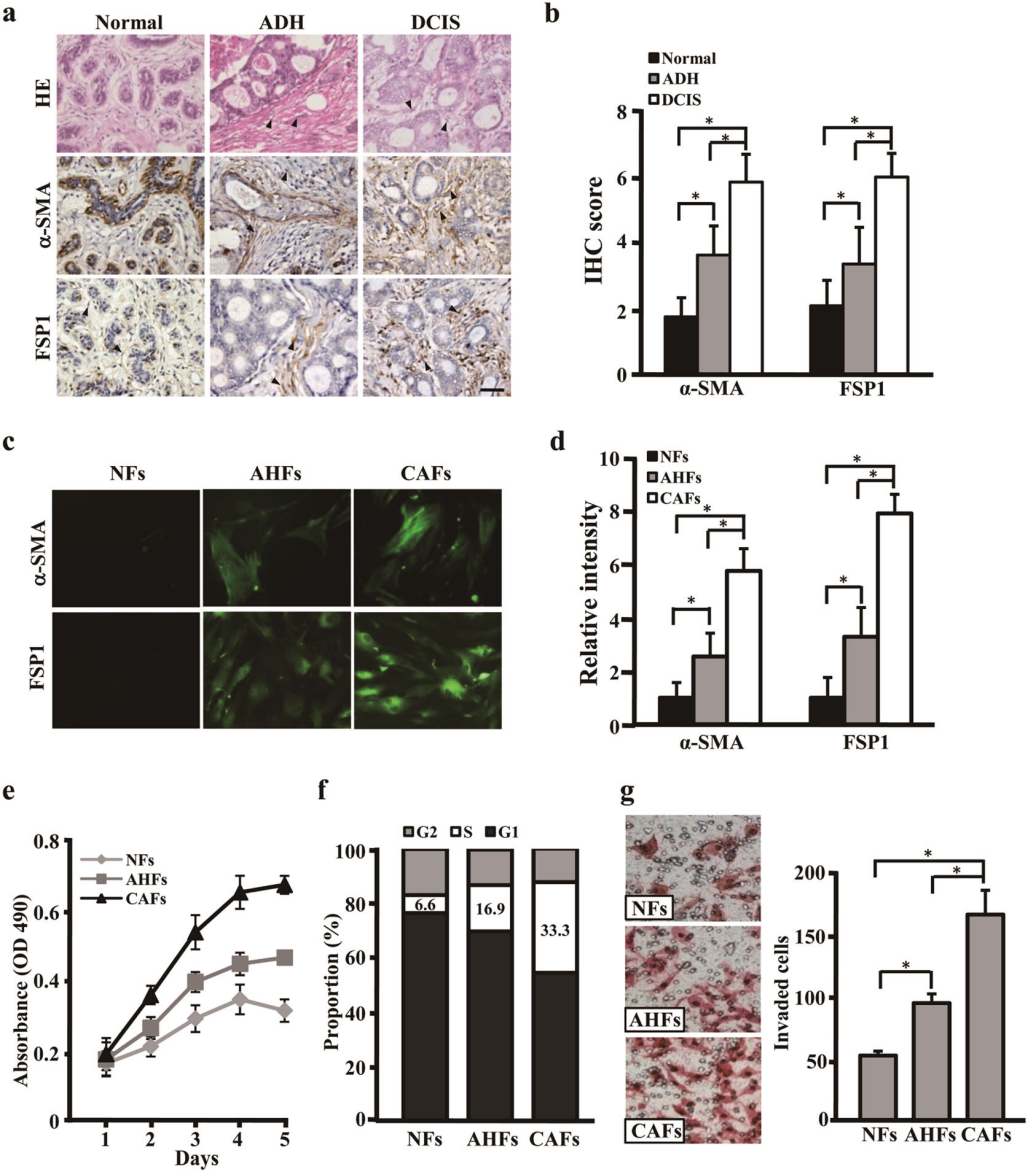
AHFs are one kind of activated myofibroblasts in mammary atypical hyperplasia tissues and possess essential biological characteristics to CAFs and NFs **a**, **b** IHC staining (**a**) and quantitation (**b**) of α-SMA and FSP1 expressions in human normal mammary tissues (normal), breast atypical ductal hyperplasia (ADH), and breast ductal carcinoma in situ (DCIS). The black arrows indicate distinctly stained fibroblast cells in the representative tissues. Scale bars, 100 μm. **c**, **d** Immunofluorescence staining (**c**) and quantitation (**d**) of α-SMA and FSP1 expressions in primary fibroblasts derived from normal mammary tissues (NFs), mammary atypical hyperplasia (AHFs), and breast ductal carcinoma in situ (CAFs). **e** Cell proliferation determined by MTT assay for NFs, AHFs, and CAFs. **f** The percentages of S-phase cells in cell cycle are shown by histogram for NFs, AHFs, and CAFs. **g** Transwell chamber analysis to determine cell invasion abilities of NFs, AHFs, and CAFs. The data were shown as mean ± SD for *N* ≥ 3 separate experiments, *p < 0.05

These evidences have suggested CAFs, an activated fibroblasts in tumor microenvironment, act critical roles to tumor growth and development^16,17^. Only a few of studies indicate that aberrant fibroblasts may exist in precancerous lesion tissues in some of tumors^18^. To answer whether a kind of activated fibroblasts in human mammary precancerous tissues, a set of mammary histopathologic frozen tissues, including 21 normal breast tissues (normal), 37 ADH, and 35 DCIS were validated by Hematoxylin and eosin (HE) staining for histopathologic grade and Immunohistochemistry (IHC) staining to identify the activated fibroblasts using their biomarkers of α-smooth muscle actin (α-SMA), fibroblast specific protein-1 (FSP1). Interestingly, we detected an amount of α-SMA- and FSP1-positive fibroblasts in ADH (we called AH fibroblasts, AHFs), which are negative in NFs and more strong staining in CAFs of DCIS (Fig. 1a and b). These findings were further confirmed by immunofluorescence staining in the freshly isolated NFs, AHFs, and CAFs (Fig. 1c and d).

In our previous studies, we found that CAFs from tumor tissues possess strong cell growth and invasion abilities than that of NFs^11,19,20^. Thus, we wondered to know the biology characteristics of AHFs. As shown in Fig. 1e–g, AHFs acquire more significant growth predominance than NFs, but notably inferior than CAFs (Fig. 1e and f). Similar invasive features were revealed (Fig. 1g). These data suggest that AHFs are one kind of activated fibroblasts and have independent biological characteristics between CAFs and NFs.

### AHFs stimulate cell growth and polarity change of epithelium-like tumor cell MCF-7 under co-culture condition

**Fig. 2:**
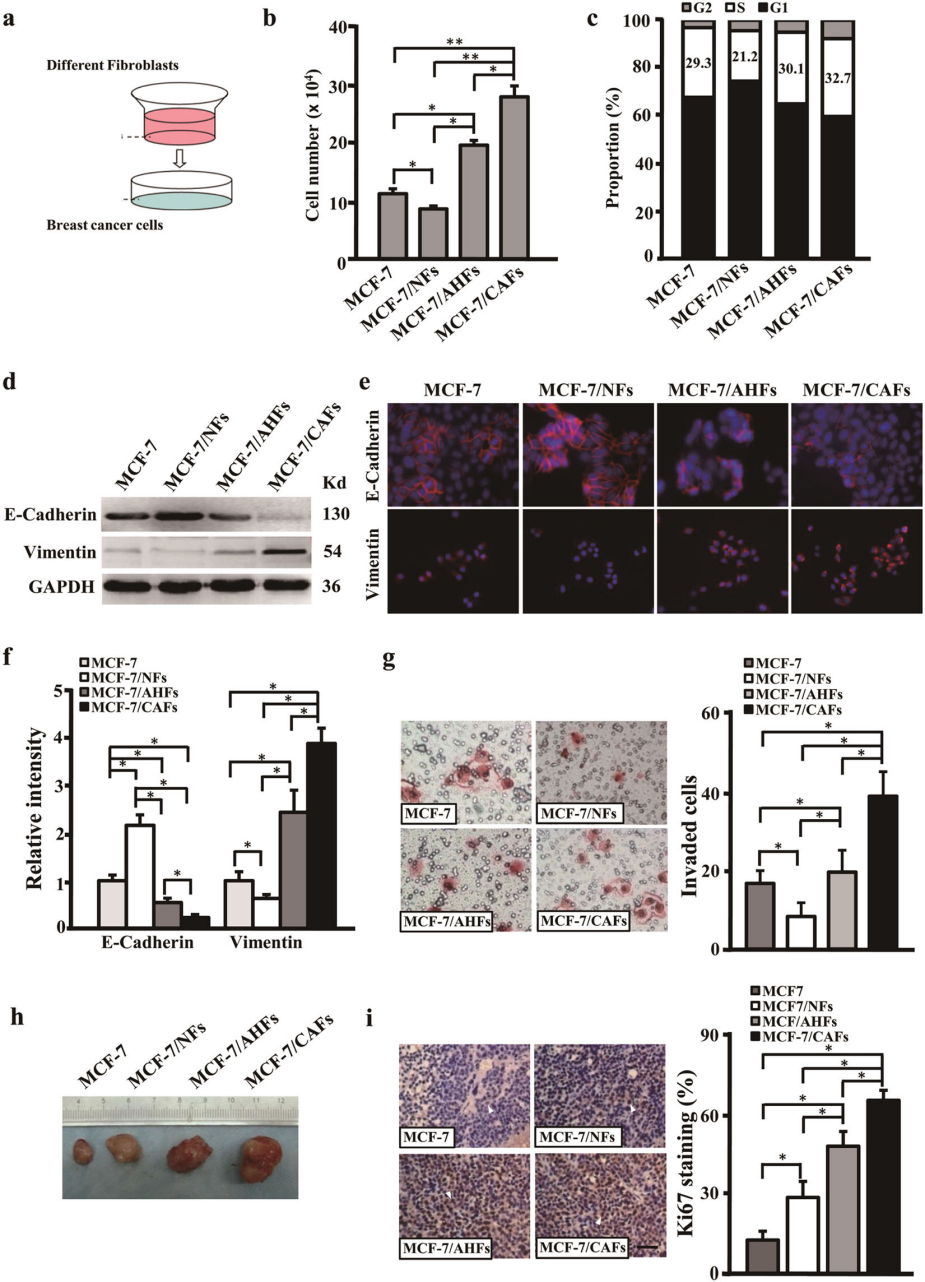
AHFs stimulate cell growth and polarity change of MCF-7 under co-culture conditions **a** A sketch to depict the 3D co-cultured system of fibroblasts and MCF-7. **b** Cell proliferation of MCF-7 was determined by cells count under co-culture system with stromal fibroblasts (NFs, AHFs, or CAFs). **c** The percentage of S-phase cells in cell cycle was shown for MCF-7 alone or MCF-7 co-cultured with NFs, AHFs, or CAFs. **d** Western blotting analysis to determine the E-Cadherin and Vimentin expression in MCF-7 or MCF-7 co-cultured with NFs, AHFs, or CAFs; GAPDH was used as a loading control. **e**, **f** Immunofluorescence staining (**e**) and quantitation (**f**) of E-cadherin and Vimentin expression in MCF-7 or MCF-7 co-cultured with NFs, AHFs, or CAFs. **g** Transwell chamber analysis to detect the invaded cells for MCF-7 or MCF-7 co-cultured with NFs, AHFs, or CAFs (magnification ×200). **h** Indicating tumor size in mice injected with MCF-7 cells alone or MCF-7 cells mixed with the indicated stromal fibroblasts. **i** Ki-67 staining in the xenograft samples. The white arrows indicate distinctly stained cells in the representative xenograft tissues. Scale bars, 100 μm. The data were shown as mean ± SD for *N* ≥ 3 separate experiments, *p < 0.05

Previous studies have shown that CAFs can promote tumor cell growth and invasion in tumor microenvironment^11,21^. We want to know whether AHFs could contribute to tumor initiation, thus a co-culture system was employed to unravel the interaction crosstalk between AHFs and tumor cells (Fig. 2a). In contrast to MCF-7 alone (MCF-7) or MCF-7 co-cultured with NFs (MCF-7/NFs), MCF-7 co-cultured with AHFs (MCF-7/AHFs) had stronger proliferation potential than that of MCF-7 and MCF-7/NFs, although it was weaker than that of MCF-7 co-cultured with CAFs (MCF-7/CAFs) tested by cell number (Fig. 2b) and cell cycle analysis (Fig. 2c). Moreover, MCF-7 co-cultured with AHFs for 2 weeks had a cell polarity changed to some extent. For example, E-cadherin, the epithelial biomarker, was decreased; and Vimentin, the mesenchymal biomarker, was increased detected by western blotting (Fig. 2d) and immunofluorescence staining (Fig. 2e and f). And the MCF-7 acquired stronger invasion ability in MCF-7/AHFs than these in culture alone or in MCF-7/NFs (Fig. 2g). In addition, the mice injected with MCF-7 in combination with AHFs (MCF-7/AHFs) had a bigger tumor than the mice injected MCF-7 alone (MCF-7) or MCF-7 mixed with NFs (MCF-7/NFs); interestingly, it was obviously smaller than the tumor generating from injection of MCF-7 mixed with CAFs (MCF-7/CAFs) (Fig. 2h and Supplementary Fig. 1). Tumor cells proliferation was assessed by Ki67 staining. Similar to the findings of AHFs and CAFs promoting cell proliferation of MCF-7 in vitro, the tumor injected with MCF-7/AHFs and MCF-7/CAFs had an enhanced Ki67 in the xenografts (Fig. 2i). These data indicate that AHFs play a role in promoting proliferation and polarity change for MCF-7, and act as oncogenic function for tumor initiation in the early tumor microenvironment, although these functions are weaker than CAFs.

### Stromal miR-200b and miR-200c are downregulated during mammary epithelium malignancy and contribute to fibroblast activity

**Fig. 3:**
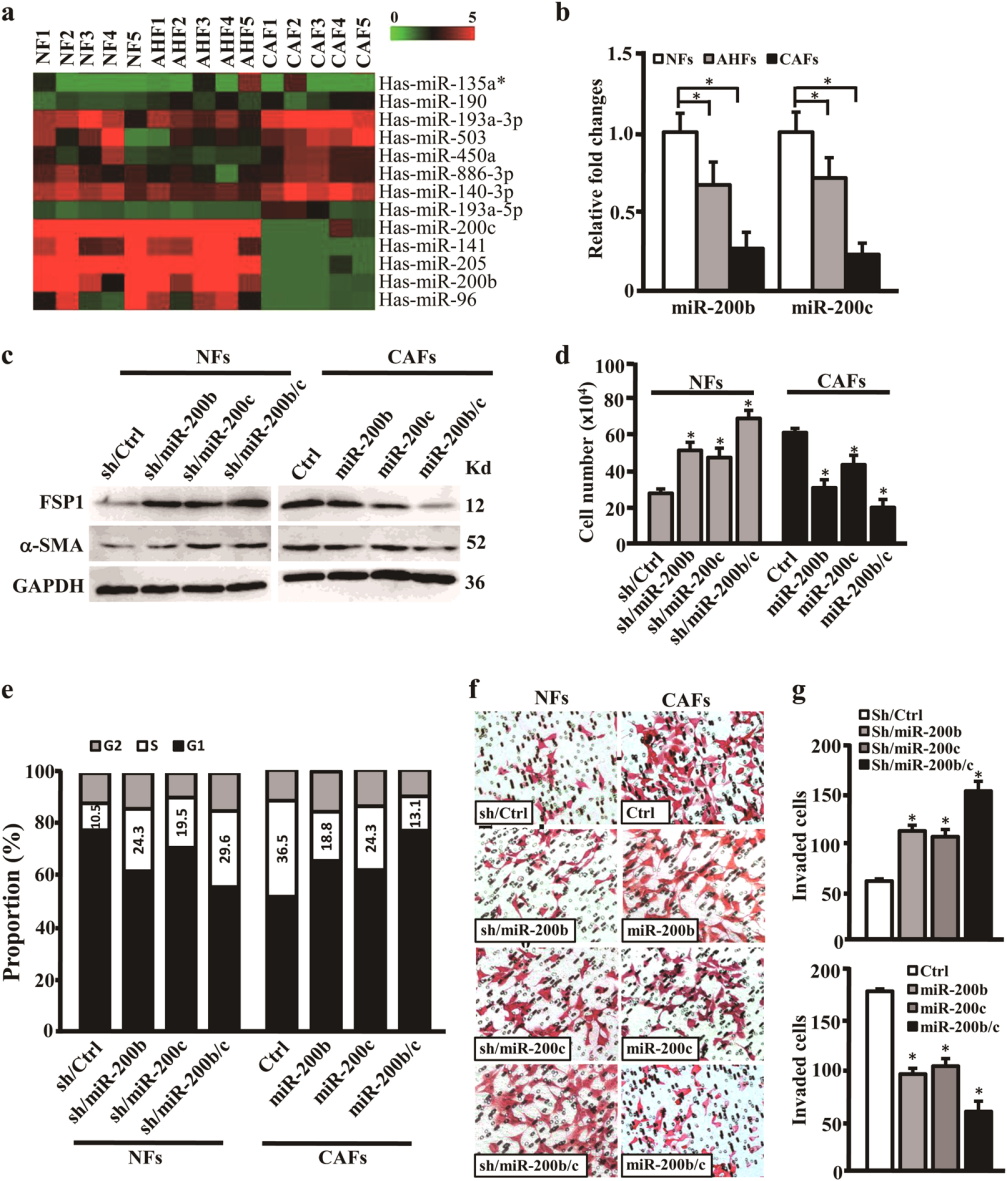
Decreased miR-200b/c induce activation of stromal fibroblasts **a** The different miRNAs among NFs, AHFs, and CAFs were identified by miRNA array analysis. **b** The expressions of miR-200b/c identified by miRNA array were confirmed using qRT-PCR in NFs, AHFs, and CAFs. U6 was used as an internal control. **c** Western blots of α-SMA and FSP1 expressions are shown in the indicated engineered CAFs or NFs; GAPDH was used as a loading control. **d**, **e** The cell count (**d**) and the percentages of S-phase cells in cell cycle (**e**) are shown by histogram in the indicated engineered CAFs or NFs. **f**, **g** Transwell chamber analysis to test the cell invasion potential of the indicated engineered CAFs or NFs (magnification ×200). The invaded cells are shown by histogram (**g**). The data were shown as mean ± SD for *N* ≥ 3 separate experiments, *p < 0.05

To uncover the potential roles of miRNAs to fibroblast activity during mammary epithelium malignancy, we performed miRNA microarray to investigate miRNAs expression profiles of NFs, AHFs, and CAFs. Compared with the miRNAs expression in NFs, nine upregulated and five downregulated miRNAs were identified in AHFs and CAFs (*p* < 0.05, *q* = 0) (Fig. 3a). Among these aberrant miRNAs, the downregulated miR-200b and miR-200c (miR-200b/c) have been reported to involve in cell differentiation and epithelium malignancy in cancer^9,22^. To confirm these findings, the gradual downregulation of miR-200b/c were further corroborated in NFs, AHFs, and CAFs freshly isolated from normal mammary tissues, ADH, and DCIS (Fig. 3b), suggesting that miR-200b/c may be the important regulators in fibroblasts activity. Next, engineered CAFs with ectopic miR-200b/c and NFs with specific short hairpin shRNA (shRNA) against miR-200b/c were established (Supplementary Fig. 2a and b). The expressions of α-SMA and FSP1 were attenuated after overexpression of miR-200b/c in CAFs (Fig. 3c). Whereas, α-SMA and FSP1 expressions were increased in NFs which knock down of miR-200b/c (Fig. 3c). To disclose the functional effects of miR-200b/c on activated fibroblasts, the cell growth, proliferation, and invasion were evaluated. As shown in Fig. 3d–g, knockdown of miR-200b/c in NFs endowed fibroblasts with vigorous proliferative potential, such as more growth rate, more S-phase cells in cell cycle (Fig. 3d and e, left panel), active mobility, and invasion (Fig. 3f, left panel; and Fig. 3g, top panel). However, overexpression of miR-200b/c in CAFs led to reduce of cell proliferation (Fig. 3d and e, right panel), and caused a marked drop of cell mobility and invasion (Fig. 3f, right panel; Fig. 3g, bottom panel). Further, the functional effects of miR-200b/c on AHFs activity were examined. Knockdown of miR-200b/c in AHFs endowed AHFs a stronger proliferative potential (Supplementary Fig. 3a, left panel) and invasive ability (Supplementary Fig. 3b, left panel; Supplementary Fig. 3c, top panel). Meanwhile, proliferative potential (Supplementary Fig. 3a, right panel) and invasive ability (Supplementary Fig. 3b, right panel; Supplementary Fig. 3c, bottom panel) of AHFs were blunted when overexpressing miR-200b/c in AHFs. Collectively, these data suggest that progressive loss of miR-200b/c induces the activation of fibroblasts, and facilitates their proliferation and invasion potentials.

### IKKβ is a critical target in miR-200b/c-mediated NF-κB activation of fibroblasts

**Fig. 4:**
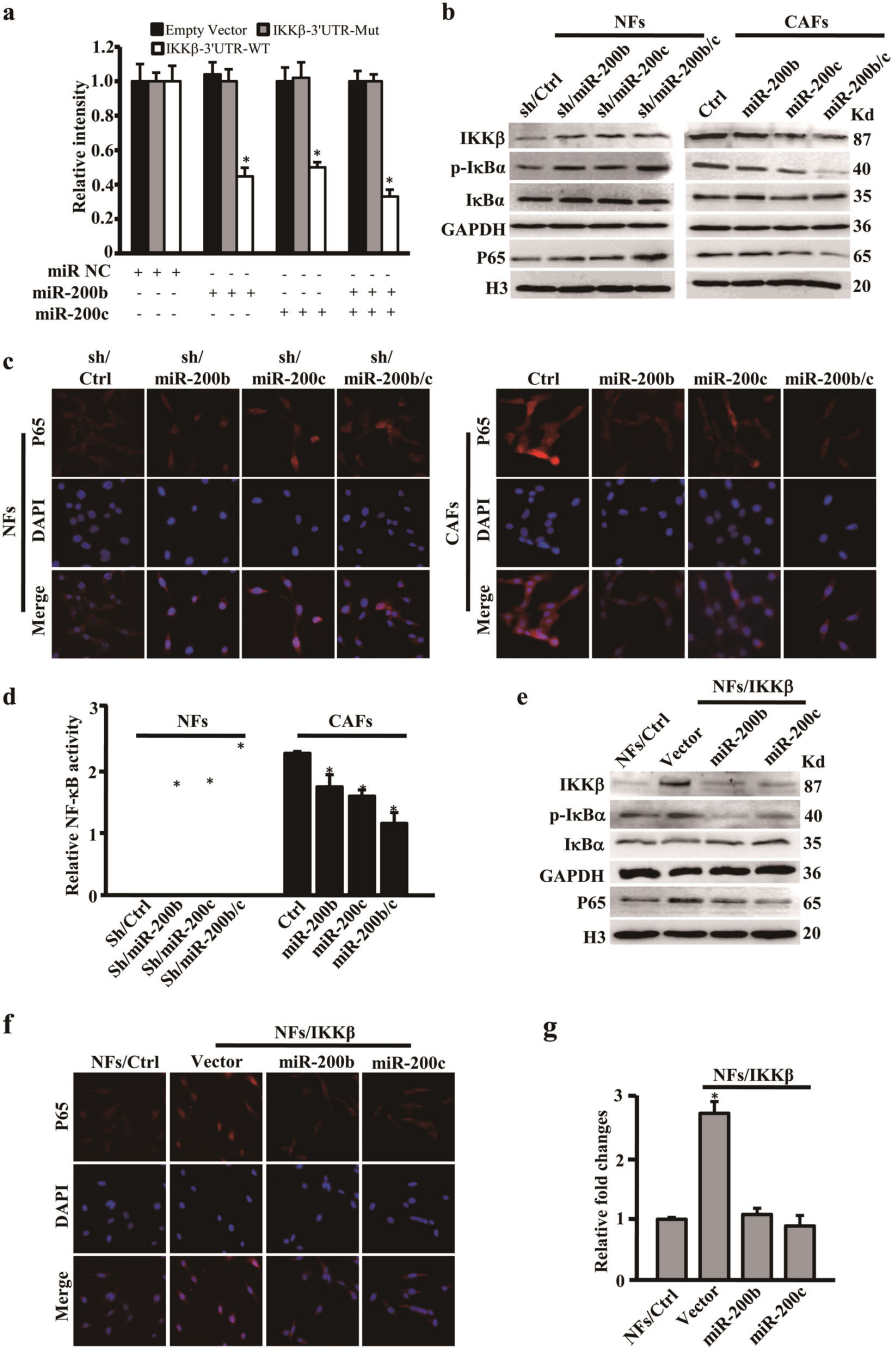
miR-200b/c and their target IKKβ induce NF-κB activation in stromal fibroblasts **a** Luciferase assay was used to test relative luciferase activities of IKKβ in 293T cells co-transfected with the indicated miR-200b/c or control vector with corresponding reports. **b** Western blotting analysis to check the protein levels of IKKβ, IκBα, p-IκBα, and nuclear P65 in the indicated engineered CAFs or NFs. GAPDH or histone 3 (H3) was used as a loading control. **c** Immunofluorescence staining of nuclear translocation of P65 in the indicated engineered CAFs or NFs (magnification ×200). **d** ELISA-based measurement of P65 activity to show NF-κB function in the indicated engineered CAFs or NFs. **e** Western blotting analysis of IKKβ, IκBα, p-IκBα, and nuclear P65 protein expressions in the NFs, NFs/IKKβ with or without miR-200b/c encoding vector. GAPDH or histone 3 (H3) was used as a loading control. **f** Immunofluorescence staining to show nuclear translocation of P65 in the NFs, NFs/IKKβ with or without miR-200b/c expression vector (magnification ×200). **g** ELISA-based measurement of P65 activity to detect NF-κB function in the NFs, NFs/IKKβ with or without miR-200b/c. The data were shown as mean ± SD for *N* ≥ 3 separate experiments, *p < 0.05

In order to find the miR-200b/c targets associated with fibroblasts activation, bioinformatics analyses (Target Scan, miRanda, and DIANA-microT) were performed. The 3′-UTR of IKKβ was complementary conserved sequences within the “seed sequences” of miR-200b/c (Supplementary Fig. 4a). Using luciferase assay, the 3′-UTR of IKKβ was consolidated to be suppressed by miR-200b/c and mutation of the binding sites in the 3′-UTR of IKKβ impaired the responsiveness of IKKβ to miR-200b/c (Fig. 4a). Furthermore, the mRNA and protein levels of IKKβ were decreased after rescue of miR-200b/c expression in CAFs, and increased in NFs after knockdown of miR-200b b/c expression (Supplementary Fig. 4b and Fig. 4b). Thus, IKKβ is directly regulated by miR-200b and miR-200c in fibroblasts.

The IKKβ is critical to NF-κB activity via phosphorylating IκBα in translocation of P65 into the nucleus^23^, indicating that loss of miR-200b/c in fibroblasts during the process of breast tumor initiation may be accompanied with activation of NF-κB. Indeed, blockage of miR-200b/c expression in NFs resulted in increased p-IκBα and nuclear P65 protein levels (Fig. 4b, left panel). Accordingly, overexpression of miR-200b/c significantly decreased p-IκBα in CAFs, which accompanied with the reduced nuclear P65 levels (Fig. 4b, right panel). Further, IKKβ, p-IκBα, and nuclear P65 protein levels in AHFs were upregulated by knockdown of miR-200b/c (Supplementary Fig. 5a, left panel), whereas were declined when miR200b/c were overexpressed in AHFs (Supplementary Fig. 5a, right panel). The miR-200b/c regulated nuclear translocation of P65 in CAFs, AHFs, and NFs was proved by immunofluorescence staining (Fig. 4c and Supplementary Fig. 5b), and NF-κB in CAFs, AHFs, and NFs activity was certified by enzyme-linked immunosorbent assay (ELISA) assay (Fig. 4d and Supplementary Fig. 5c).

To address whether IKKβ involving in miR-200b/c regulated NF-κB activation in stomal fibroblasts, the engineered NFs with ectopic IKKβ (Supplementary Fig. 4c) was used to detect translocation of P65. As expectedly, stable expression of IKKβ enhanced p-IκBα and nuclear P65 levels in NFs, whereas miR-200b or miR-200c mimics repressed p-IκBα and nuclear P65 proteins in the engineered NFs/IKKβ cells (Fig. 4e). Similarly, Immunofluorescence staining and ELISA assay also strengthened that IKKβ is a key mediator in miR-200b/c regulated NF-κB activation (Fig. 4f and g). Together, these data show that IKKβ is indispensable for the miR-200b/c-mediated NF-κB activation in fibroblasts.

### NF-κB activity plays a core role in fibroblasts activation

**Fig. 5:**
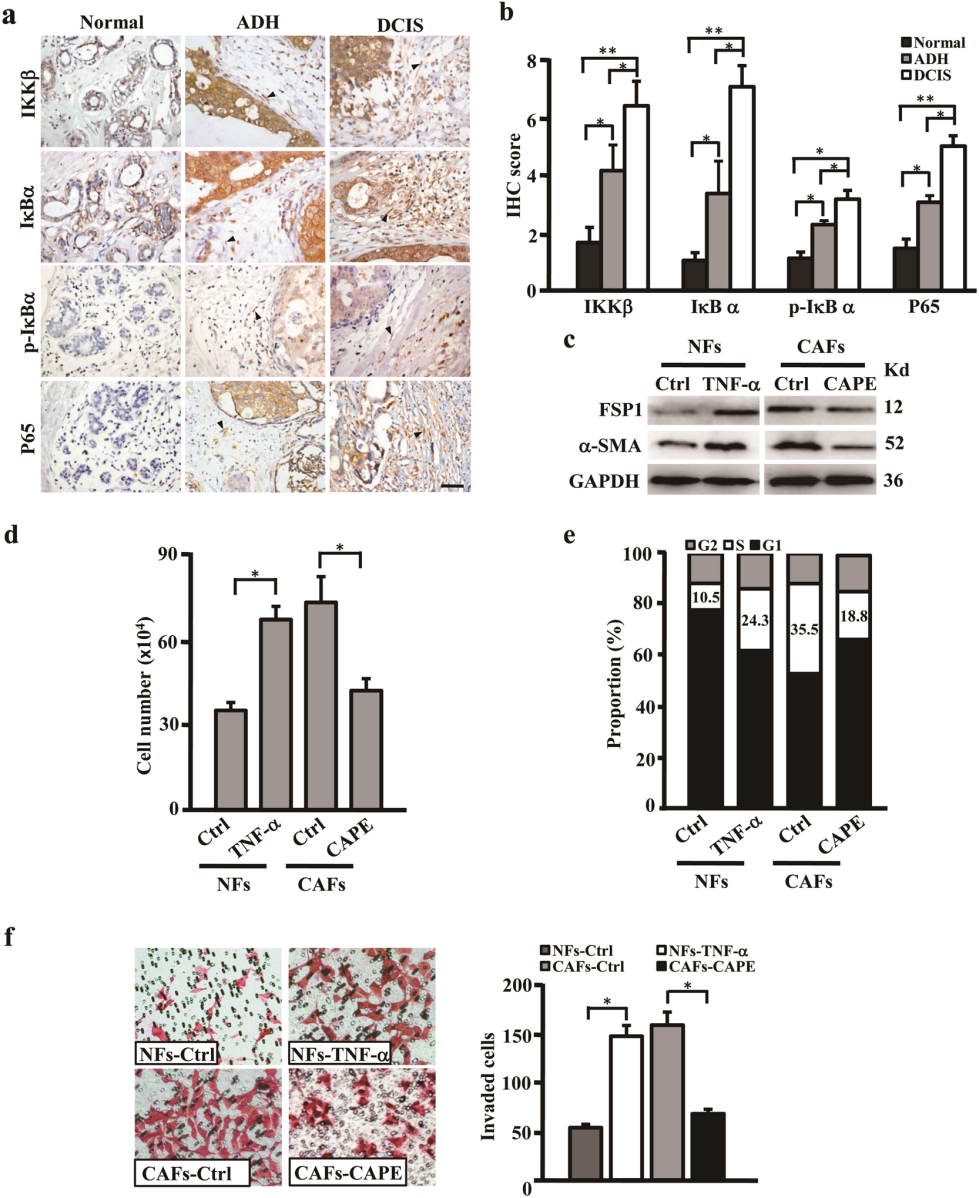
NF-κB enhances stromal fibroblasts activation **a**, **b** IHC staining (**a**) and quantitation (**b**) of IKKβ, IκBα, p-IκBα, and nuclear P65 protein levels in human normal mammary tissues (normal), breast atypical ductal hyperplasia (ADH), and breast ductal carcinoma in situ (DCIS). The black arrows indicate distinctly stained fibroblasts in the representative tumor tissues. Scale bars, 100 μm. **c** Western blotting analysis of α-SMA and FSP1 expressions in NFs treated with or without TNF-α and CAPE. GAPDH was used as a loading control. **d** and **e** The cell count (**d**) and the percentages of S-phase cells in cell cycle (**e**) were shown by histogram in NFs treated with or without TNF-α and CAPE. **f** Transwell chamber analysis for detection of the cell invasion of NFs treated with or without TNF-α and CAPE. The data were shown as mean ± SD for *N* ≥ 3 separate experiments, *p < 0.05. TNF-α tumor necrosis factor-α, CAPE caffeic acid phenethyl ester, NF-κB inhibitor

Next, we asked whether NF-κB is a key promoting factor to fibroblasts activation during the process of breast tumor initiation. Thus, the expressions of IKKβ, IκBα, p-IκBα, and P65 were detected in normal, ADH, and DCIS tissues by immunohistochemistry staining (Fig. 5a). As expectedly, the levels of IKKβ, p-IκBα and nuclear P65 were progressively increased from normal tissues to ADH and DCIS (Fig. 5b). Activation of NF-κB in NFs stimulated by adding exogenous TNF-α (tumor necrosis factor-α, one of the known activator to NF-κB), α-SMA, and FSP1 expressions of NFs were increased (Fig. 5c). Correspondingly, the cell proliferation (Fig. 5d and e) and invasion abilities (Fig. 5f), the representative biologic characteristics of activated fibroblasts, were increased in response to NF-κB activation stimulated by TNF-α. However, inhibition of NF-κB signaling in CAFs using CAPE, an inhibitor of NF-κB, α-SMA, and FSP1 protein levels were decreased (Fig. 5c) in CAFs. Cell proliferation (Fig. 5d and e) and invasion (Fig. 5f) of CAFs were severely blunted after losing NF-κB activation. These data support that NF-κB activity induces the activated fibroblasts in the process of breast tumor initiation.

### PAI-1 mediates the crosstalk between the activated fibroblasts and epithelium-like tumor cell MCF-7 to promote tumor cell proliferation and cell polarity change

**Fig. 6:**
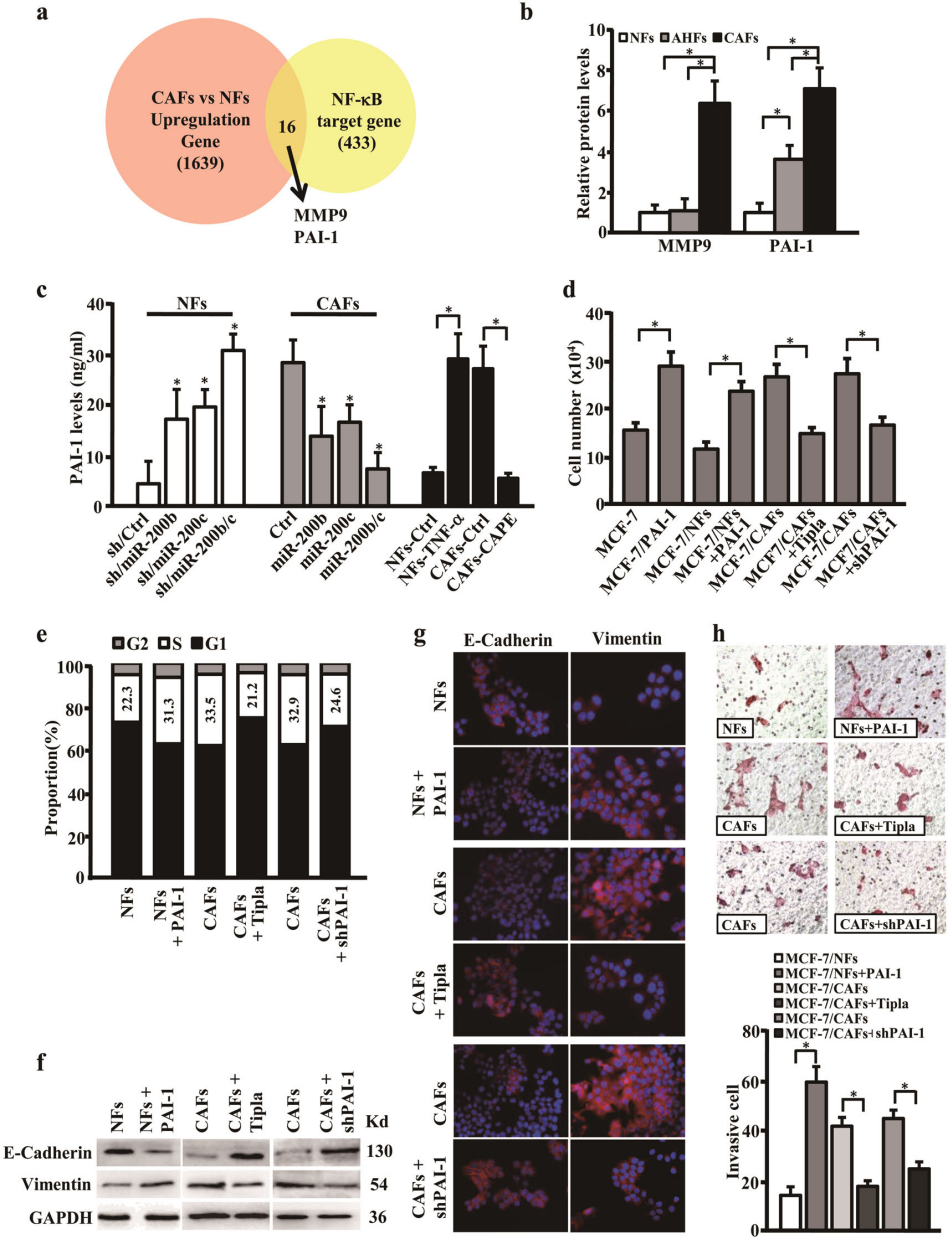
PAI-1 derived from activated stromal fibroblasts promotes tumor cell proliferation and cell polarity change of MCF-7 **a** Venn diagram to show the identified target genes and the cytokines regulated by NF-κB in our published microarray data. **b**, **c** ELISA analysis to determine the secreting protein levels of MMP9 and PAI-1 in NFs, AHFs, and CAFs (**b**), or PAI-1 in the indicated fibroblasts (**c**). **d** MCF-7 cells were cultured alone or co-cultured with supernatant derived from NFs or CAFs under treatment as shown. The MCF-7 cell amounts are shown by histogram. The used concentration of PAI-1:50 µM; tiplaxtinin (Tipla): 30 µM. **e** Histogram to show the percentages of S-phase cells in cell cycle for MCF-7 co-cultured with supernatant derived from NFs or CAFs under the treatment as shown. The used concentration of PAI-1 and tiplaxtinin are as in **d**. **f**–**h** Cell culture and treatments of MCF-7 cells are as same as described in **e**. Western blotting analysis (**f**) and Immunofluorescent staining (**g**) to detect E-Cadherin and Vimentin expressions in MCF-7. Cell invasion of MCF-7 (**h**) was analyzed by Transwell chamber analysis (magnification ×200). The data were shown as mean ± SD for *N* ≥ 3 separate experiments, *p < 0.05. MMP9 matrix metalloproteinase 9, PAI-1 plasminogen activator inhibitor-1, Tiplaxtinin (Tipla) PAI-1 inhibitor

CAFs have been suggested to promote cancer cell progression by secreting cytokines and chemokines^24^. We sought to determine whether the activated fibroblasts could influence MCF-7 proliferation and cell polarity. After analyzing the database of NF-κB target genes (NF-κB Transcription Factors Database, Boston University)^25^ and the aberrant genes in our previous microarray data of CAFs^11^, 16 of interesting targets, including PAI-1 and MMP9 (Matrix metalloproteinase 9), which could induce cells growth and invasion^26,27^, were founded (Fig. 6a and Supplementary Table 3). After confirmation of these target expressions in NFs, AHFs, and CAFs using quantitative real-time PCR (qRT-PCR) and ELISA, PAI-1 was revealed to be progressively increased from NFs to AHFs and CAFs (Supplementary Fig. 6a and Fig. 6b). Furthermore, secreting PAI-1 was significantly enhanced in the supernatant from the NFs which miR-200b/c were silenced by specific shRNAs or NF-κB was stimulated by TNF-α; and reduced in the supernatant from CAFs which miR-200b/c were rescued by overexpression or NF-κB was blocked using inhibitor CAPE (Fig. 6c); the expression levels of PAI-1 in AHFs were also regulated by altering miR-200b/c levels or NF-κB activation (Supplementary Fig. 7a). These data suggest that miR-200b/c and NF-κB closely regulate PAI-1 expression in the activated fibroblasts.

Our above findings disclosed that activated fibroblasts could endow MCF-7 with strong cell proliferation potential and induce cell polarity change under co-culture system (Fig. 2), we wondered whether PAI-1 involved in the crosstalk between fibroblasts and MCF-7 and plays a critical role to biologic changes of tumor cells. As shown in Fig. 6d, compared with the MCF-7 alone, addition of the recombinant PAI-1 into the supernatant of MCF-7 (MCF-7/PAI-1) could notably increase cell proliferation of MCF-7; similarly, application of the recombinant PAI-1 into the supernatant of NFs, or PAI-1 inhibitor tiplaxtinin (Tipla) into the supernatant of CAFs, or knockdown of PAI-1 expression by the specific shRNA in CAFs (Supplementary Fig. 6b), MCF-7 in the co-culture system acquired a powerful proliferation potential by PAI-1 or clearly reduced their proliferation ability after loss of PAI-1 or inhibition of PAI-1 activation in CAFs (Fig. 6d and e), respectively. Furthermore, adding recombinant PAI-1 into the supernatant of AHFs stimulated cell proliferation MCF-7; silencing PAI-1 expression or inhibition of PAI-1 activation using shRNA or tiplaxtinin obviously declined proliferation of MCF-7 in the co-culture system (Supplementary Fig. 7b and c). The cell polarity of MCF-7 was also changed revealed by western blotting analysis and immunofluorescence staining. Compared to the control supernatant, addition of PAI-1 to the supernatant from NFs resulted in decreased E-cadherin and enhanced Vimentin levels in MCF-7 (Fig. 6f, left panel, Fig. 6g, upper panel), and conferred MCF-7 an invasive advantage (Fig. 6h, upper panel). On the other hand, loss of PAI-1 (using shRNA) or inhibition of PAI-1 activity (using tiplaxtinin) in supernatant from CAFs increased E-cadherin and mitigated Vimentin expression in MCF-7 (Fig. 6f, middle and right panels, Fig. 6g, middle and bottom panels), thus receded MCF-7 invasive ability in co-cultured with CAFs (Fig. 6h, middle and bottom panels). Similarly, addition of PAI-1 to the supernatant from AHFs destroyed the cell polarity of MCF-7 and promoted invasion of MFC-7; and loss of PAI-1 or inhibition of PAI-1 activity in supernatant from AHFs had a benefit to maintain the cell polarity of MCF-7 and reduced its cell invasion (Supplementary Fig. 7d–f). These results suggest that PAI-1 mediates the crosstalk between activated fibroblasts and epithelium-like tumor cell MCF-7 and promotes tumor cell proliferation and cell polarity change.

## Discussion

During breast tumor initiation and progression, the mammary microenvironment undergoes a number of dynamic and regulated alterations that occur in parallel to transformation^13^. Previous studies reported that CAFs contribute to breast cancer development, but CAFs are hardly explained by the dynamic process of primed fibroblasts in premalignant microenvironment. In this study, AHFs are identified and isolated from ADH, should be a better evidence for discussing the dynamic changes of fibroblasts in premalignant transformation. We show that expressions of CAFs markers, α-SMA, and FSP1, are increased in AHFs and CAFs. More significantly, the growth and invasion abilities of AHFs are falling in between NFs and CAFs. In premalignant microenvironment, AHFs may be a kind of activating fibroblasts (or early stage of CAFs), which is different from quiescent NFs and activated CAFs. Thus, these activating AHFs have their unique biological characteristics between CAFs and NFs.

The specification and differentiation of mammary epithelium may be orchestrated by highly regulated contextual signals derived from the microenvironment^28^. For example, CAFs can provide tumor-promoting environments, whereas NFs are thought to suppress tumor progression^8,29^. Given the critical role of fibroblasts in mammary development, localized breast tumor, and invasive cancer, it is likely that fibroblasts are also involved in progression of benign disease to carcinoma. In this study, we found that epithelium-like MCF-7 could obtain stronger proliferation ability when they were co-cultured with AHFs and CAFs but not NFs. Importantly, MCF-7 co-cultured with AHFs and CAFs lose their E-cadherin, and gain Vimentin and high potential of cell invasion. These effects were obviously weaker when MCF-7 grown with NFs under the same experimental conditions. Compared with NFs, AHFs exhibit an increased propensity to induce the growth and polarity changes for MCF-7, although these capacities are not enough strong as well as CAFs. Our findings indicate that fibroblasts in premalignant microenvironment are involved in premalignant transformation of mammary epithelium and play a role in control the malignant behavior of epithelial cells.

The miRNA profiles between NFs and CAFs are different and several features of CAFs phenotype are attributable to miRNA dysregulation^30^. Although many miRNAs are identifid as CAFs related, little is known which miRNA contributes to activation of NFs into CAFs. The miR-200 family consisting of five members (miR-200a, −200b, −200c, −141, and −429) is an emerging miRNA family that has been shown to play crucial roles in cancer progression^22^. Recent research done in our laboratory reveal that miR-200 families (miR-200a, −141, −200b, and −200c) are generally downregulated in activated CAFs, and act as direct mediators for NFs reprogramming into CAFs and ECM remodeling^9^. In this study, miR-200b/c are gradually diminishing from NFs to AHFs and CAFs, which play a role to influence the early predisposed AHFs into activated CAFs. Importantly, NFs knocked down with miR-200b/c could be primed and differentiated into CAF-like fibroblasts, which have the characteristics of enhanced α-SMA and FSP1, and strong growth and invasion abilities. Our data suggest that the activated transformation of fibroblasts from normal to active status is accompanied by a gradual downregulation of miR-200b/c.

The dimeric NF-κB transcription factor, whose subunits belong to the Rel family of DNA-binding proteins, plays a critical role in immune and inflammatory responses^23^. Although the involvement of innate and adaptive immune cells in cancer-promoting inflammation is well established, studies have implicated fibroblasts in this process^31^. CAFs promote tumor growth by expression of the proinflammatory signature, and induction and maintenance of this proinflammatory signature are NF-κB dependent^31,32^. In our study, miR-200b/c-mediated inhibition of IKKβ partially explains the effects of the miR-200b/c on the IκB phosphorylation and NF-κB activation in activated fibroblasts. In the meantime, we determine the effects of NF-κB activation on the activated transformation of fibroblasts. Hence, miR-200b/c and IKKβ/NF-κB play a key role in fibroblasts activation.

Plasminogen activator inhibitor-1 (PAI-1) as a secreting protein is shown to effect tumor cell adhesion, migration, and invasion^33,34^. Several reports show that PAI-1 expression is under the control of NF-κB^35,36^, and CAFs are the major PAI-1-positive cells in invasive ductal breast carcinomas^37^. Here, we find that PAI-1 expression increases from NFs to AHFs and CAFs. Furthermore, secreting PAI-1 is regulated by miR-200b/c and NF-κB activation in fibroblasts. CAFs have been shown to play a significant role in promoting breast cancer progression and metastasis through paracrine signaling^38^. In our study, we reveal that PAI-1 secreted from activated fibroblasts AHFs and CAFs regulates cell growth and polarity changes of epithelium-like tumor cell MCF-7. When loss of PAI-1 or inhibiting PAI-1 activity in the co-culture system, MCF-7 co-cultured with CAFs or AHFs lack a powerful proliferation potential and renew cell polarity. These studies suggest that a close crosstalk between fibroblasts in the premalignant stage of breast tumor and PAI-1 expression has a critical role to malignant behavior of mammary epithelium.

In summary, our study suggests an important role of AHFs that induce and support tumor initiation and progression. The activation of fibroblasts is driven by decreased miR-200b and miR-200c, which trigger malignant behavior of epithelial cells growth and polarity changes. It is a novel evidence for uncovering the mechanisms behind fibroblasts during the malignant transformation, initiation, and tumor progression.

## Materials and methods

### Tissue samples

All of human breast tissues were obtained with approval from the First Affiliated Hospital of the Chongqing Medical University, Chongqing, China. Fresh surgical specimens (mastectomies and minimally invasive surgery) were available from patients with DCIS, ADH, and normal breast tissue. Hematoxylin and eosin (H&E)-stained frozen sections were prepared from each tissue sample to confirm the information about histological subtype and histopathological grade. All patients had not previously undergone radiotherapy or chemotherapy treatment.

The study was approved by the Ethics Committee of Chongqing Medical University, and was conducted in compliance with the Helsinki Declaration. All patients involved in this study consented to participate in the study and publication of its results.

### Fibroblast isolation and cell culture

Primary fibroblast cells were isolated and immortalized using human TERT as described previously^20^. The primary NFs, fibroblasts in the AHFs, the fibroblasts of DCIS (CAFs), and the paired immortalized CAFs and NFs were routinely maintained in DMEM (Invitrogen,Carlsbad, CA, USA) containing 10% FBS at 37 °C in humidified atmosphere containing 5% CO_2_. The three to six passage of primary fibroblast cells was used in the experiments.

### miRNA and mRNA microarray analysis

Total RNA was isolated from CAFs, AHFs, and NFs using the mir-VanaTM miRNA isolation Kit (Ambion, Austin, TX, USA) following the manufacturer’s instructions. Probe synthesis and hybridization to Agilent Human microRNA Microarray v2.0 (Agilent, Santa Clara, CA, USA) were performed by using the miRNA complete labeling and hybridizing Kit (Agilent) following protocols recommended by the producer. Analysis of the arrays was performed using the GeneSpring GX v10.0 (Agilent) and R statistics package (R v2.14.0) as described previously^15^.

The mRNA microarray data obtained our previous microarray data of CAFs and NFs. Paired SAM analysis was applied as described previously^11^.

### Plasmid construction, inhibitors, and mimics

pLenti4.1-puro-pri-miR-200b and pri-miR-200c were constructed as described previously^9^. pLenti4.1-puro-IKKβ vector was purchased from GeneChem (Shanghai, China). The synthetic shRNA oligonucleotides (Invitrogen) specifically against the *miR-200b/200c*, *IKKβ*, or *PAI-1* genes were inserted into the pLVX-shRNA1 lentivector (Clontech, Palo Alto, CA, USA). To generate the luciferase reporters of the direct targeting IKKβ by miR-200b/c, the synthetic oligonucleotides corresponding to the wild-type- and mutant-binding sites of miR-200b/c in the 3′-UTR of IKKβ were inserted into the Spe 1 and Hind III sites of pMIR-Report vector (Ambion). Mimics and inhibitors of miR-200b/c and the corresponding controls were the products of GenePharma. The oligonucleotides are listed in Supplementary Table 1.

### Co-culture experiments

In co-culture system, 1 × 10^4^ primary fibroblasts (NFs, AHFs, or CAFs) were seeded into the Boyden chamber inserter with 0.4 μm pore size (Corning Inc., NY, USA), MCF-7 cells (3 × 10^4^ cells) were seeded into the bottom well. The co-culture system was maintained in DMEM with 1% FBS, and the co-culture medium was changed every 3 days at 50%. According to different experiments, recombinant protein and/or inhibitors were separately added in the co-cultures medium, including caffeic acid phenethyl ester (CAPE) (5 µM, selleckchem, TX, USA), tiplaxtinin (30 µM, selleckchem), recombinant TNF-α (50 ng/ml, Abcam), and recombinant PAI-1 (50 µM, Pepro Tech, NJ, USA). For cell proliferation assay, MCF-7 cells in the co-culture system were maintained for 3 days; for western blotting analysis or immunofluorescence, the MCF-7 cells were co-cultured with fibroblasts and with or without recombinant protein, and/or inhibitors for 2 weeks.

### Cell invasion assay

Fibroblasts invasion assay was measured using the transwell assay as described previously^20^. Briefly, 1 × 10^4^ fibroblasts (CAFs, AHFs, NFs, or engineered fibroblasts) in 200 μl serum-free medium were seeded into the Boyden chambers of 8 mm pore size (Corning) coated with ECM (1:7.5) (Sigma). FBS medium of 10% was separately added into the bottom chamber. After 8 h of incubation, the invaded cells on the opposite side of the filter were counted.

In order to determine the invasion of MCF-7 cells in co-culture with fibroblasts, 2 × 10^4^ MCF-7 cells in 200 μl serum-free medium were seeded into the upper well of the Boyden chambers coated with ECM. The fibroblasts (CAFs, AHFs, NFs, or engineered fibroblasts; 1 × 10^4^ cells per well) were cultured in the well of the supplied 24-well platein serum-free medium with or without exogenous CAPE, tiplaxtinin, recombinant TNF-α, and PAI-1. After co-culture for 24 h, the invaded MCF-7 cells on the opposite side of the filter were counted. All data represent at least three experiments and done in triplicate (mean ± SD).

### Immunohistochemistry and immunofluorescence

Fixed with 10% buffered formalin, the paraffin-embedded specimens were sectioned at 4 μm thickness and stained with H&E according to standard histopathological techniques.

Sections of tissues were examined by immunohistochemical staining with antibodies using a previously reported method^20^. CAFs, AHFs, NFs, or MCF-7 cells were fixed within 4% paraformaldehyde at room temperature. After washing with PBS, cells were treated by 0.1% triton-100 and incubated with 5% goat serum solution at 37 °C to block nonspecific interactions. Then cells were stained with specific antibodies against α-SMA (1:200; Abcam), FSP1 (1:150; Abcam), P65 (1:400; Abcam), E-cadherin (1:150; Bioworld), or Vimentin (1:400; Abcam). Normal rabbit IgG was used as a negative control. A fluorescein isothiocyanate (FITC)-labeled goat anti-rabbit IgG was used as secondary antibody. Sections were then mounted in aqueous medium containing DAPI as a nuclear counterstain.

### Western blot analysis

The dissected cortical tissues and cultured cells were homogenized in cold RIPA lysis buffer. Nuclear extracts from the cortex were obtained using nuclear and cytoplasmic protein extraction Kit (Beyotime, Shanghai, China). Equal amounts of proteins (50 μg) were loaded into SDS-PAGE (8, 10, or 12%), electrophoresed, and transferred onto PVDF membranes (Millipore, Temecula, CA). Nonspecific binding sites were blocked by incubating with 5% non-fat milk, and then the membranes were incubated with primary antibodies. Antibodies against α-SMA (1:1000; Abcam), FSP1 (1:800; Abcam), E-cadherin (1:800; Bioworld), Vimentin (1:4000; Abcam), IKKβ (1:1000; Abcam), IκBα (1:1000; Abcam), P65 (1:5000; Abcam), p-IκBα (1:2000; RabMAb), H3 (1:2000; RabMAb), and GAPDH (1:5000; Abcam). Immunoreactive bands were visualized with the enhanced chemiluminescence (ECL) chemiluminescence system (Millipore). GAPDH or H3 was used as loading control for cytoplasmic or nuclear proteins. The bands were semi-quantified using ImageJ software. All data represent at least three experiments and done in triplicate (mean ± SD).

### Quantitative real-time PCR

Total RNA was isolated using Trizol® (Invitrogen) according to the manufacturer’s instructions. RNA was subjected to reverse transcription reactions by using the PrimeScript RT reagent Kit (Takara Bio, Dalian, China). Quantitative real-time PCR was performed by CFX Connect™ real-time PCR detection system (Bio-Rad, Hercules, CA, USA) using SYBR^®^ Premix Ex Taq™ II (Takara Bio). Relative fold changes of gene expression were calculated by the ΔΔCT method and the values are expressed as 2^−ΔΔCt^. The primers used in qRT-PCR are listed in Supplementary Table 2. All experiments were performed at least three times (mean ± SD).

### Cell proliferation and flow cytometric analysis

Fibroblasts growth was tested with 3-(4,5-dimethylthiazol-2yl)-2,5-diphenyl tetrazolium bromide (MTT) assay as previously described^20^. A density of 3 × 10^4^ cells per well were seeded into 96-well plate with 200 μl of complete growth medium. MTT (5 mg/ml) was added to each well and incubated for 4 h in 37 °C. After addition of 0.1 ml of isopropanol with 100 μl DMSO to each well, absorbance was measured using an ultraviolet spectrophotometric reader at a wavelength of 490 nm. The independent experiments were repeated for five wells (mean ± SD).

The proliferation assay of MCF-7 cells co-cultured with fibroblasts or engineered fibroblasts were counted via a Biorad^®^ TC20™ automated cell counter. The S-phase cells in cell cycle were analyzed by flowcytometry using standard methods. A minimum of 20,000 events was collected to maximize the statistical validity of the compartmental analysis. The experiments were done in triplicate and repeated three times (mean ± SD).

### Enzyme-linked immunosorbent assay

Conditioned media from the co-culture system or from 1 × 10^6^ fibroblasts (NFs, AHFs, or CAFs) were harvested, and the concentrations of NF-κB (P65) activity (TransAM p65 kit, Active Motif, CA, USA), PAI-1 (Boster Bio, Hubei, China), and MMP9 (Boster Bio) were measured by ELISA kit following the manufacturer’s instructions. The absorbance (450 nm) of each sample was analyzed using a standard ELISA plate reader. The experiments were repeated for five wells (mean ± SD).

### Luciferase reporter assay

A total of 1 × 10^5^ CAFs were seeded in 24-well plates and co-transfected with miR-200b/c mimics or miRNA control (miRNA NC), and pMIR-IKKβ (wild-type or mutant) or the control plasmid pRL-TK (Promega, Madison, WI, USA) using Lipofectamine 2000 (Invitrogen). After culture for 48 h, cells were lysed and collected, and Renilla and luciferase activities were measured with a Dual-Luciferase Reporter System (E1910, Promega). The experiments were done in triplicate and repeated three times (mean ± SD).

### Xenograft models

To determine cell tumorigenicity in vivo, 4 × 10^6^ MCF-7 cells mixed with 2 × 10^6^ primary fibroblasts (CAFs, AHFs, or NFs) were injected into mammary fat pads of female Balb/c nu/nu mice (*n* = 5), which was performed accordance with guidelines on animal care and use established by the Chongqing Medical University Experimental Animal Management Committee. Same amount of MCF-7 cells injected alone were the controls (*n* = 5). Tumor growth was determined by measuring the two axes of the tumor every 7th day. Mice were killed when tumors reached 1.5–2 cm in diameter. The tissues were fixed in 4% paraformaldehyde and processed for immunohistochemistry to detect Ki67 (1:200, bioworld). Tumor volume was calculated using the ellipsoid formula: volume = 1/2 × *a* × *b*^2^ (*a* = length, *b* = width).

### Statistical analysis

Statistical analysis measurements are presented as means ± SD comparisons; student’s *t* test (two groups), and one-way analysis of variance (three or more groups) with Student–Newman–Keuls multiple comparisons test were performed using GraphPad Prism 6 statistical packages (GraphPad Software, San Diego, CA, USA). A probability value of *p* < 0.05 was considered to indicate statistical significance.

## Electronic supplementary material

Supplementary Figure legends

supplementary figure 1-7

Supplementary Tables1-3
