# Supplementary material for: Primed atypical ductal hyperplasia-associated fibroblasts promote cell growth and polarity changes of transformed epithelium-like breast cancer MCF-7 cells via miR-200b/c-IKKβ signaling
Source: Cell Death Dis. 2018 Jan 26;9(2):122. doi: 10.1038/s41419-017-0133-1 (PMC5833401; doi:10.1038/s41419-017-0133-1)
Supplement: Supplementary file 1 — Supplementary Figure legends [file 41419_2017_133_MOESM1_ESM.doc]

**Supplementary Figure legends**

**Supplementary Figure 1 Tumor growth curves.** MCF-7 cells mixed with the indicated stromal fibroblasts were subcutaneously injected into nude mice in nude mice (n=5 per group), tumor growth was measured.

**Supplementary Figure 2 Expression of miR-200b and miR-200c in the engineered fibroblasts. (a)** qRT-PCR analysis of the expression of miR-200b and miR-200c in CAF/miR-200b and CAF/miR-200c cells. **(b)** qRT-PCR analysis of the expression of miR-200b and miR-200c in NFs-sh/miR-200b and NFs-sh/miR-200c cells. U6 was used as an internal control. The data are shown as mean±SD for N=3 separate experiments.

**Supplementary Figure 3 miR-200b and miR-200c regulate the activation of AHFs.** **(a)** The percentages of S-phase population of AHFs with over-expressing miR-200b/c or knocked-down of miR-200b/c in cell cycle were shown by histogram. **(b** and **c)** Transwell chamber analysis to test the cell invasion potentials of AHFs over-expressing miR-200b/c or knocked-down of miR-200b/c (magnification 200x). The invaded cells are shown by histogram (c). The data are shown as mean±SD for N≥3 separate experiments, **p*< 0.05.

**Supplementary Figure 4 IKKβ  is a target gene of miR-200b/c and expresses in the engineered fibroblasts. (a)** Diagrammatic representation of the predicted miR-200b/c binding sites in the IKKβ 3’-UTRs. **(b)** qRT-PCR was used to determine IKKβ  expression in the indicated engineered CAFs or NFs. β-Actin was used as a loading control. **(c)** qRT-PCR analysis of the expression of IKKβ in NFs/Ctrl and NFs/IKKβ cells. The data are shown as mean±SD for N=3 separate experiments, **p*< 0.05.

**Supplementary Figure 5 miR-200b and miR-200c induce NF-κB activation in AHFs. (a)** Western blot analysis to check the protein levels of IKKβ, IkBα, p-IkBα and nuclear P65 in AHFs with over-expressing miR-200b/c or knocked-down of miR-200b/c. GAPDH or histone 3 (H3) was used as a loading control. **(b)** Immunofluorescent staining of nuclear translocation of P65 in AHFs with over-expressing miR-200b/c or knocked-down of miR-200b/c (magnification200x). **(c)** ELISA-based measurement of P65 activity toshow NF-κB functionin the indicated engineered AHFs. The data are shown as mean±SD for N≥3 separate experiments, **p*< 0.05.

**Supplementary Figure 6 Expression of PAI-1 in the indicated** **fibroblasts. (a)** qRT-PCR analysis of the expression of MMP9 and PAI-1 in NFs, AHFs and CAFs. **(b)** qRT-PCR to determine PAI-1 expression in CAFs transfected with three shRNAs against PAI-1. β-Actin was used as an internal control. The data are shown as mean±SD for N≥3 separate experiments, **p*< 0.05. MMP9: Matrix metalloproteinase 9; PAI-1: Plasminogen activator inhibitor-1;

**Supplementary Figure 7 PAI-1 derived from AHFs promotes tumor cell proliferation and cell polarity change of MCF-7. (a)** ELISA analysis to determine the secreting protein levels of PAI-1 in the indicated fibroblasts. **(b** and **c)** The cell count (b) and percentages of S-phase population in cell cycle (c) are shown by histogram for MCF-7 co-cultured with supernatant derived from AHFs under treatment of PAI-1 (50 µM), Tiplaxtinin (30 µM), or shPAI-1. **(d-f)** MCF-7 cells were co-cultured with supernatant as described in Figure b and c. Western blot analysis (d) and immunofluorescent staining (e) to detect E-Cadherin and Vimentin expressions in MCF-7; cell invasion of MCF-7 (f) was analyzed by Transwell chamber analysis (magnification 200x). The data are shown as mean±SD for N≥3 separate experiments, **p*< 0.05. CAPE: Affeic acid phenethyl ester, NF-κB inhibitor; Tiplaxtinin: PAI-1 inhibitor.
