# Supplementary figures and images for: Primed atypical ductal hyperplasia-associated fibroblasts promote cell growth and polarity changes of transformed epithelium-like breast cancer MCF-7 cells via miR-200b/c-IKKβ signaling
Source: Cell Death Dis. 2018 Jan 26;9(2):122. doi: 10.1038/s41419-017-0133-1 (PMC5833401; doi:10.1038/s41419-017-0133-1)

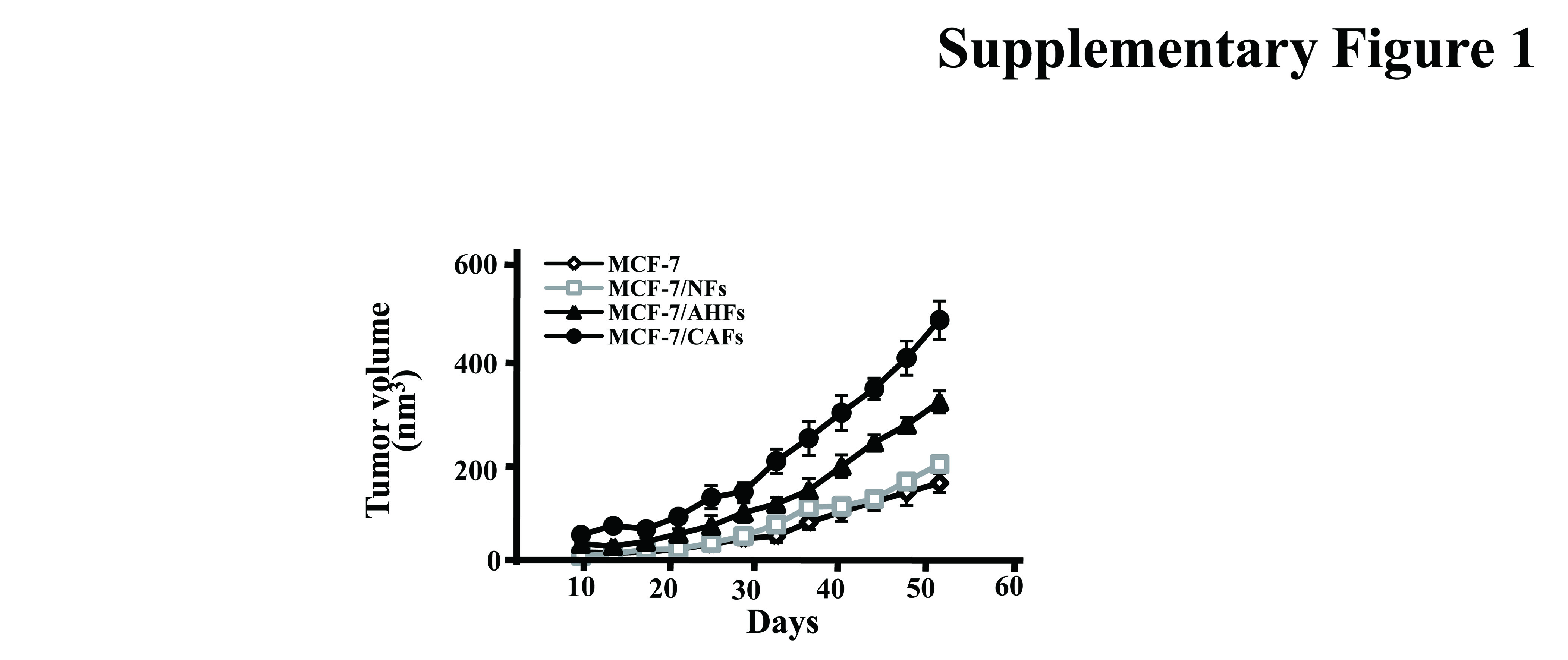


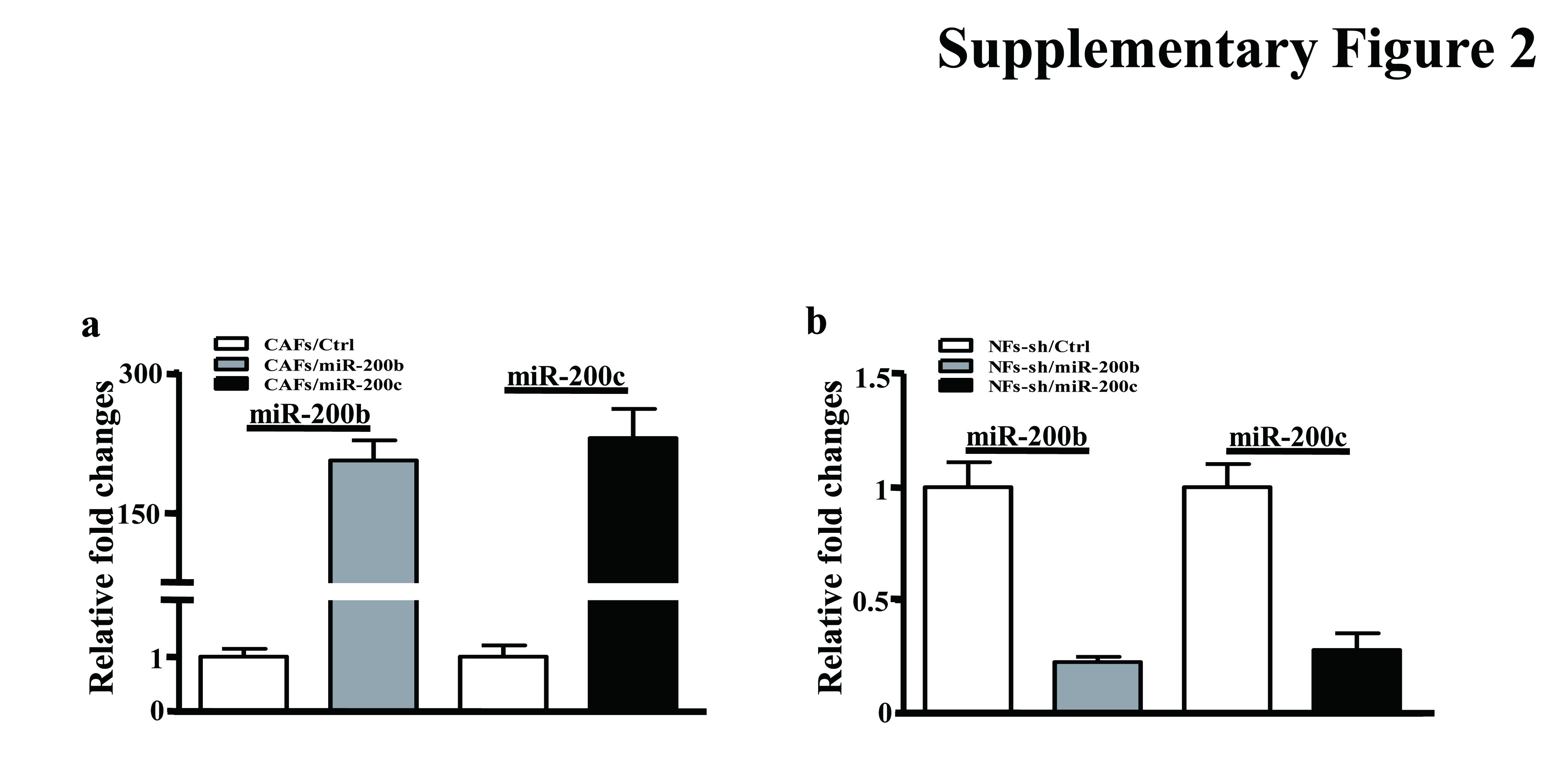


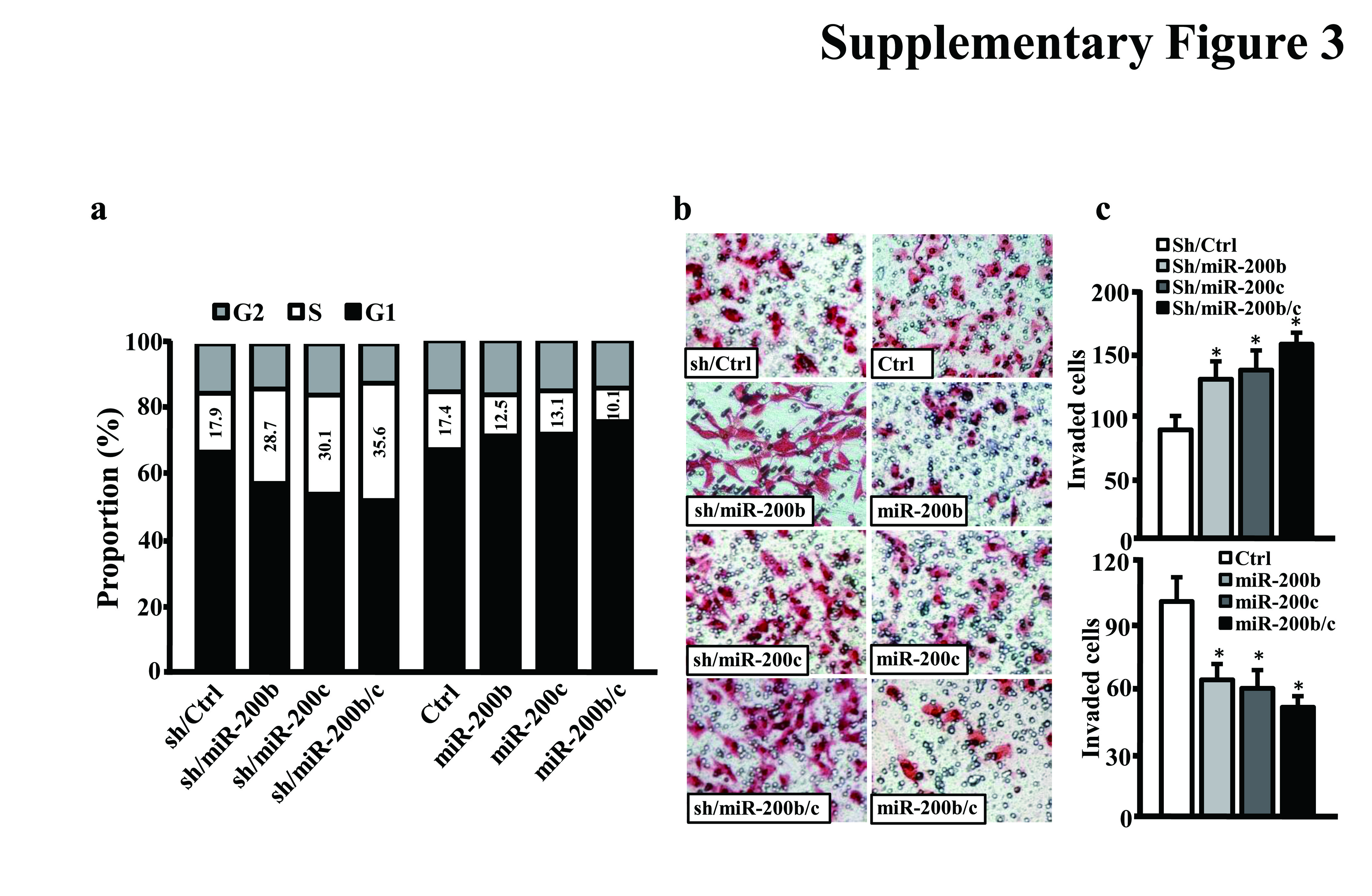


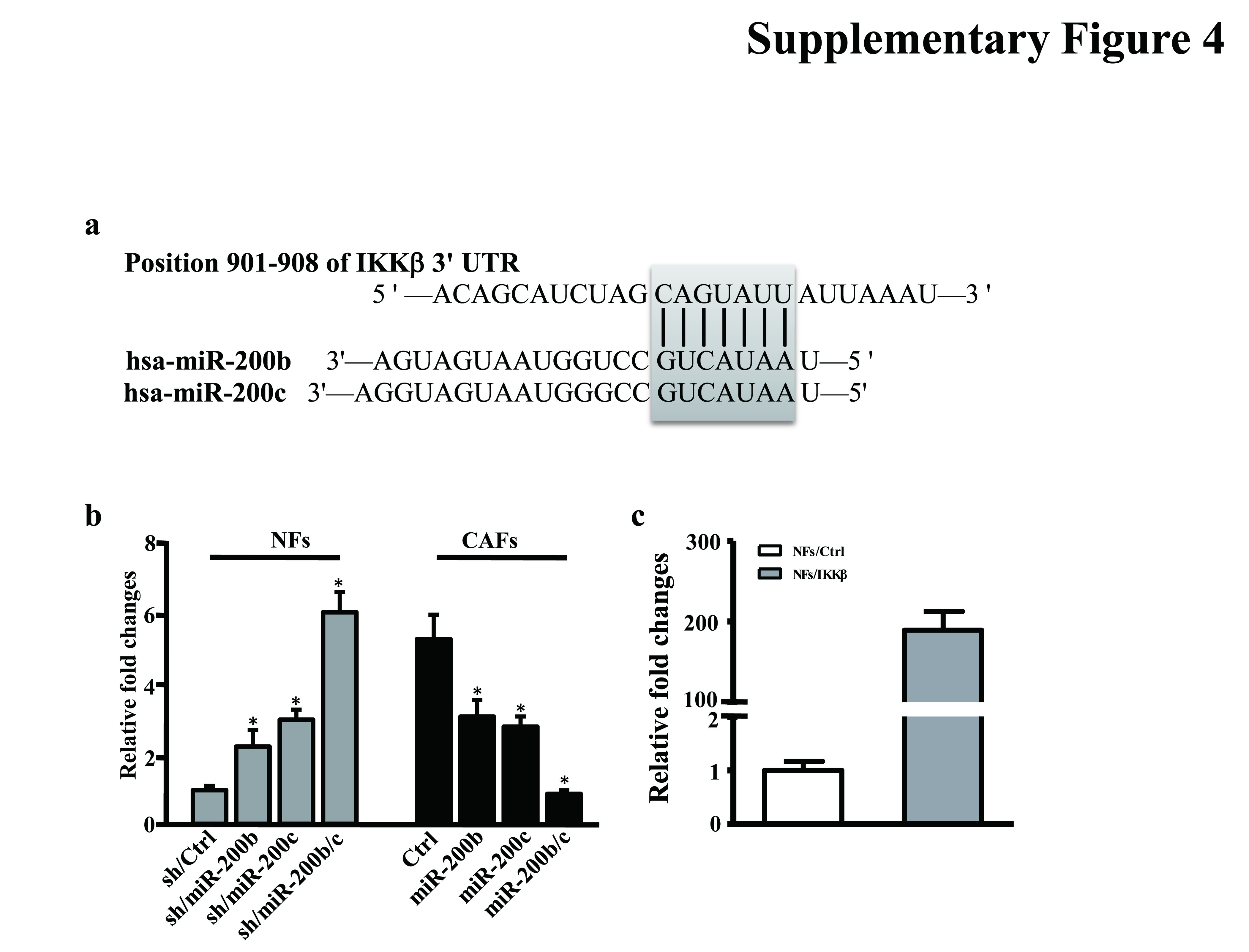


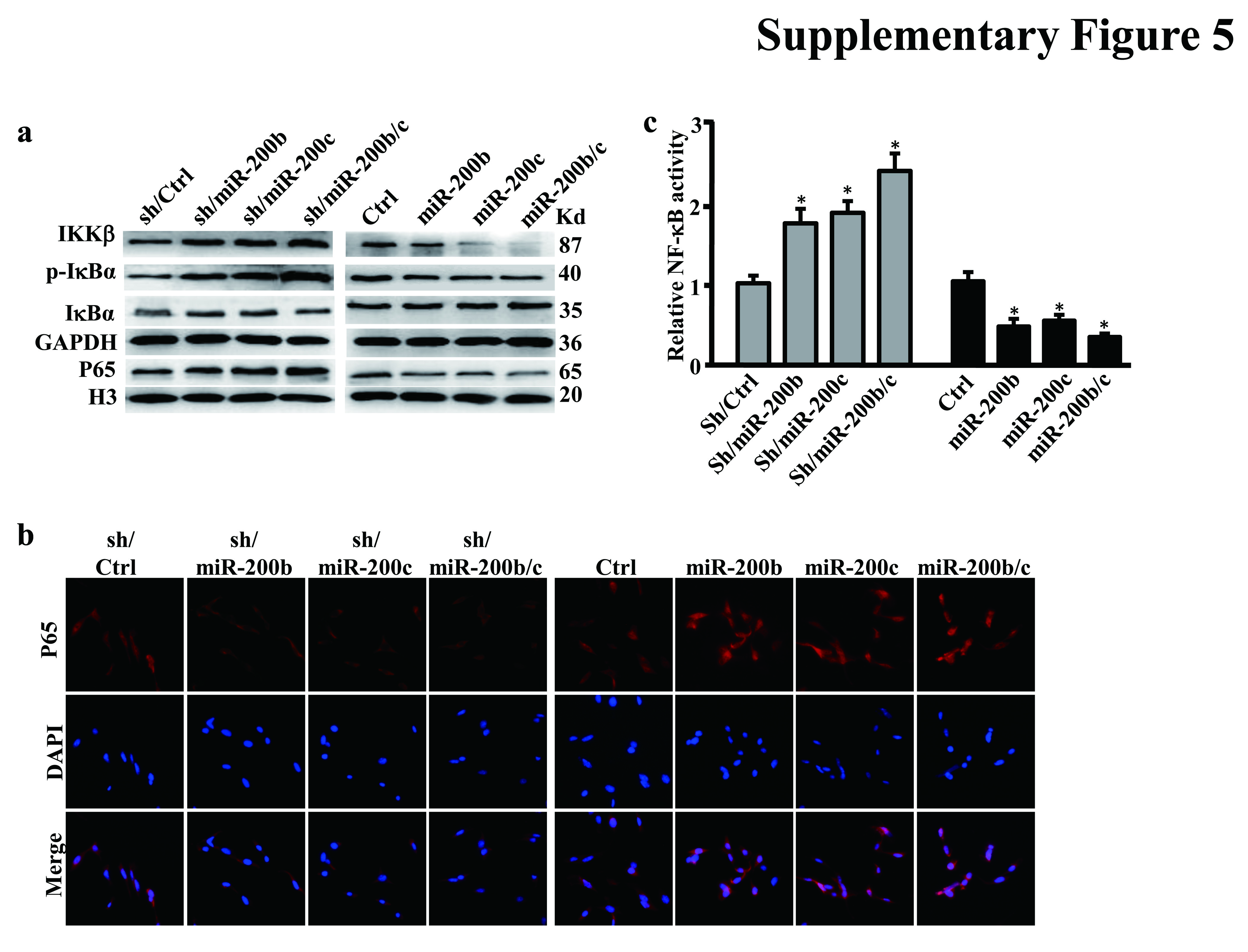

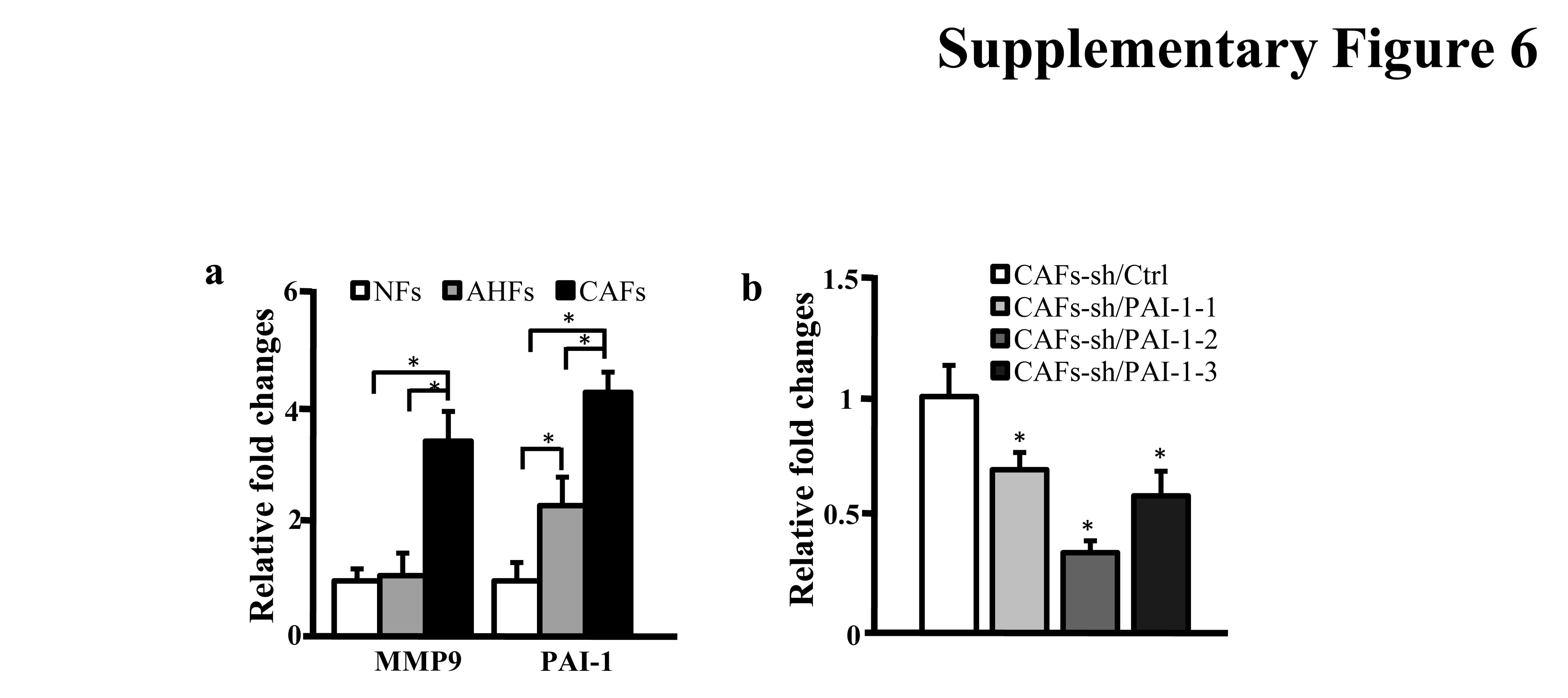

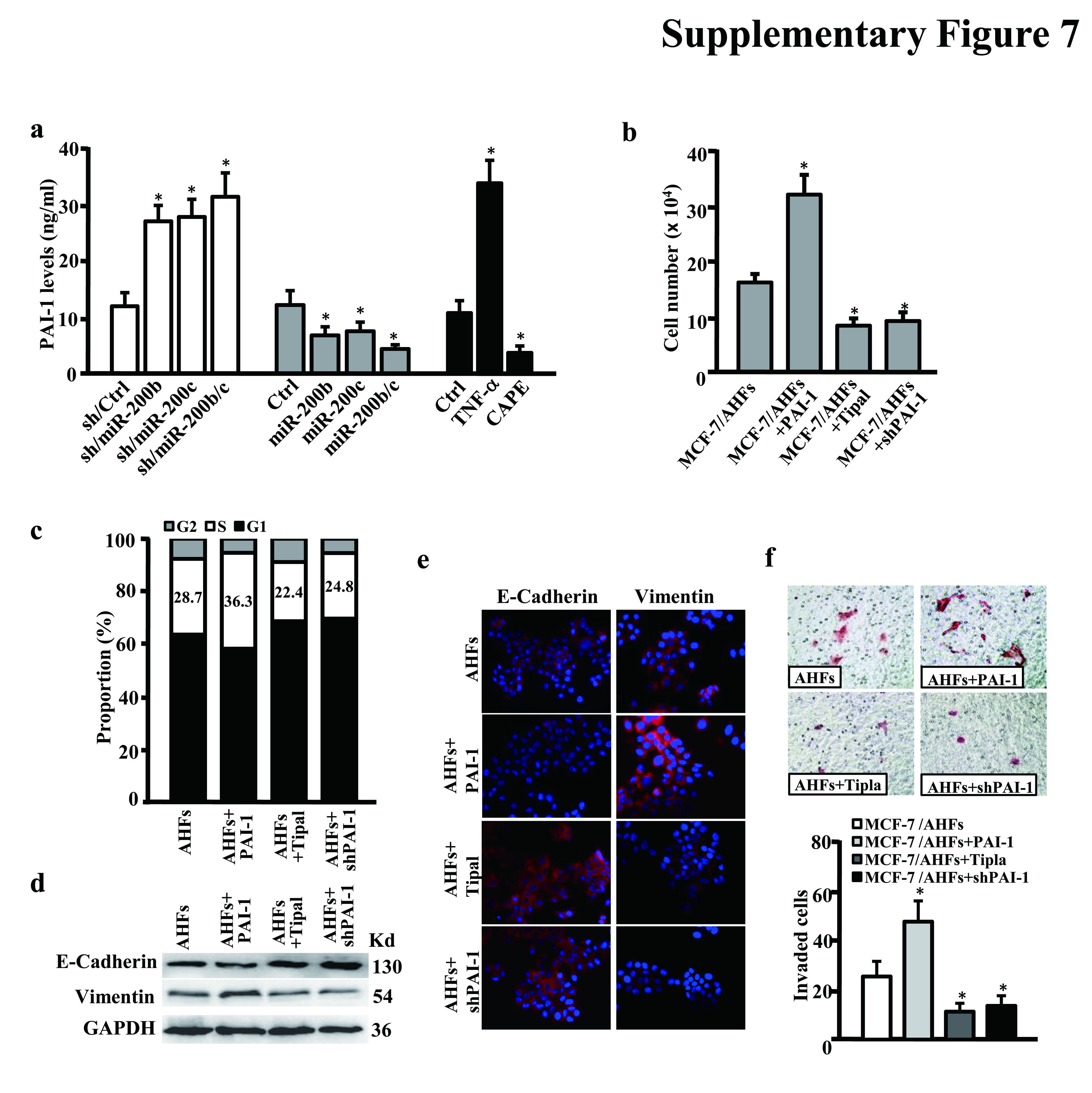

Supplement: Supplementary file 2 — supplementary figure 1-7 [file 41419_2017_133_MOESM2_ESM.docx]
