# Supplementary material for: Primed atypical ductal hyperplasia-associated fibroblasts promote cell growth and polarity changes of transformed epithelium-like breast cancer MCF-7 cells via miR-200b/c-IKKβ signaling
Source: Cell Death Dis. 2018 Jan 26;9(2):122. doi: 10.1038/s41419-017-0133-1 (PMC5833401; doi:10.1038/s41419-017-0133-1)
Supplement: Supplementary file 3 — Supplementary Tables1-3 [file 41419_2017_133_MOESM3_ESM.doc]

| **Supplementary Table 1 shRNA special against genes** | | |
| --- | --- | --- |
| **Genes** |  | **Sequence** |
| PAI-1 | F | GCCACTGGAAAGGCAACATGACGAATCATGTTGCCTTTCCAGTGGC |
|  | R | GCCACTGGAAAGGCAACATGATTCGTCATGTTGCCTTTCCAGTGGC |
| miR-200b | F | TCCAATGCTGCCCAGTAAGATG |
|  | R | CATCTTACTGGGCAGCATTGGA |
| miR-200c | F | CCAAACACTGCTGGGTAAGACG |
|  | R | CGTCTTACCCAGCAGTGTTTGG |

| **Supplementary Table 2 Primers for RT-PCR used in the experiments** | | |
| --- | --- | --- |
| **Genes** |  | **Sequence** |
| miR-200b | RT | GTCGTATCCAGTGCAGGGTCCGAGGTATTCGCACTGGATACGACTCATCA |
|  | F | CGCCTAATACTGCCTGGTAATG |
| miR-200c | RT | GTCGTATCCAGTGCAGGGTCCGAGGTATTCGCACTGGATACGACTCCATC |
|  | F | GCCTAATACTGCCGGGTAATGA |
| Universal | R | GTGCAGGGTCCGAGGT |
| U6 | F | CTCGCTTCGGCAGCACA |
|  | R | AACGCTTCACGAATTTGCGT |
| IKKβ | F | CTGGCCTTTGAGTGCATCAC |
|  | R | CGCTAACAACAATGTCCACCT |
| MMP9 | F | TCCCTGGAGACCTGAGAACC |
|  | R | GGCAAGTCTTCCGAGTAGTTT |
| PAI-1 | F | AGTGGACTTTTCAGAGGTGGA |
|  | R | GCCGTTGAAGTAGAGGGCATT |
| β-Actin | F | TGACGTGGACATCCGCAAAG |
|  | R | CTGGAAGGTGGACAGCGAGG |

| **Supplementary Table 3 16 of interesting NF-κB target genes in CAFs/NFs** | | | |
| --- | --- | --- | --- |
| **Gene Symbol** | **CAFs/NFs** | **Fold Change** | **p-value** |
| LYZ | up | 24.86 | 0.0004 |
| CD48 | up | 12.00 | 0.0004 |
| MMP9 | up | 8.46 | 0.0074 |
| CD86 | up | 6.24 | 0.0055 |
| CCL4 | up | 3.01 | 0.0432 |
| PGLYRP1 | up | 2.77 | 0.0378 |
| ADORA1 | up | 2.25 | 0.0114 |
| HIF1A | up | 2.14 | 0.0290 |
| AHCTF1 | up | 2.05 | 0.0246 |
| HSP90AA1 | up | 1.89 | 0.0325 |
| PAI-1 | up | 1.83 | 0.0398 |
| ENG | up | 1.78 | 0.0183 |
| AMACR | up | 1.62 | 0.0211 |
| REL | up | 1.55 | 0.0480 |
| SELS | up | 1.48 | 0.0088 |
| HMGN1 | up | 1.39 | 0.0261 |
